# How are evidence and knowledge used in orthopaedic decision-making? Three comparative case studies of different approaches to implementation of clinical guidance in practice

**DOI:** 10.1186/s13012-018-0771-4

**Published:** 2018-05-31

**Authors:** Amy Grove, Aileen Clarke, Graeme Currie

**Affiliations:** 1 Division of Health Sciences, Warwick Medical School, University of Warwick, Coventry, UK; 2 Entrepreneurship & Innovation, Organising Healthcare Research Network, Warwick Business School, University of Warwick, Coventry, UK

**Keywords:** Guidelines, Implementation, Orthopaedic surgery, Evidence-based medicine, Comparative case study

## Abstract

**Background:**

The uptake and use of clinical guidelines is often insufficient to change clinical behaviour and reduce variation in practice. As a consequence of diverse organisational contexts, the simple provision of guidelines cannot ensure fidelity or guarantee their use when making decisions. Implementation research in surgery has focused on understanding what evidence exists for clinical practice decisions but limits understanding to the technical, educational and accessibility issues. This research aims to identify where, when and how evidence and knowledge are used in orthopaedic decision-making and how variation in these factors contributes to different approaches to implementation of clinical guidance in practice.

**Methods:**

We used in-depth case studies to examine guideline implementation in real-life surgical practice. We conducted comparative case studies in three English National Health Service hospitals over a 12-month period. Each in-depth case study consisted of a mix of qualitative methods including interviews, observations and document analysis. Data included field notes from observations of day-to-day practice, 64 interviews with NHS surgeons and staff and the collection of 121 supplementary documents.

**Results:**

Case studies identified 17 sources of knowledge and evidence which influenced clinical decisions in elective orthopaedic surgery. A comparative analysis across cases revealed that each hospital had distinct approaches to decision-making. Decision-making is described as occurring as a result of how 17 types of knowledge and evidence were privileged and of how they interacted and changed in context. Guideline implementation was contingent and mediated through four distinct contextual levels. Implementation could be assessed for individual surgeons, groups of surgeons or the organisation as a whole, but it could also differ between these levels. Differences in how evidence and knowledge were used contributed to variations in practice from guidelines.

**Conclusion:**

A range of complex and competing sources of evidence and knowledge exists which influence the working practices of healthcare professionals. The dynamic selection, combination and use of each type of knowledge and evidence influence the implementation and use of clinical guidance in practice. Clinical guidelines are a fundamental part of practice, but represent only one type of evidence influencing clinical decisions. In the orthopaedic speciality, other distinct sources of evidence and knowledge are selected and used which impact on how guidelines are implemented. New approaches to guideline implementation need to appreciate and incorporate this diverse range of knowledge and evidence which influences clinical decisions and to take account of the changing contexts in which decisions are made.

**Electronic supplementary material:**

The online version of this article (10.1186/s13012-018-0771-4) contains supplementary material, which is available to authorized users.

## Background

Across the world, policymaking organisations exist to produce clinical guidance and recommendations for healthcare, which are based on scientific evidence. In the UK, these organisations are the National Institute for Health and Care Excellence (NICE) and the Scottish Intercollegiate Guidelines Network (SIGN) [1, 2]. Together, they provide evidence-based guidance on the most effective ways to diagnose, treat and prevent poor health for National Health Service (NHS) patients. One of the aims of clinical guidance is to reduce variation in practice, and therefore, limit inequalities in service delivery [3]. However, previous research has established that healthcare organisations face several challenges to implementation [4–6].

In 2014, a review of systematic reviews explored factors which influence guideline implementation and uptake [7]. The findings report a range of factors including those associated with guidelines themselves (e.g., complexity), with healthcare professionals (e.g., lack of awareness) and with the working environment such as limited time, personnel and resources devoted to support guideline adherence [7]. Limited information was provided to describe the context of guideline implementation in detail or the differences in healthcare environments which might impact implementation, such as differences in the processes of healthcare delivery and national policy decisions. Evidence exists describing the barriers to implementation of clinical guidance and the rates of guideline uptake across a range of diseases [3–5, 8, 9]. For example, lack of time was identified as the most commonly reported barrier to implementation [5]. However, too few studies rigorously assess the effectiveness of approaches to improve implementation of clinical guidelines or explicitly describe factors which enable their uptake and use [6, 7]. Rates of guideline uptake provide a proxy for guideline implementation, but fail to demonstrate the realistic uptake and actual use of clinical guidelines in real world healthcare practice.

There is a need for empirical research which examines a wide range of contextual characteristics influencing the uptake and use of clinical guidelines. This includes the professional and environmental factors which have been described previously, but also requires a description of the local practice context and wider healthcare sector in sufficient detail to facilitate more effective guideline implementation. Therefore, the aim of this research was to identify where, when and how evidence and knowledge are used in healthcare decision-making and how variation in these factors contributes to different approaches to implementation of clinical guidance in practice.

### Over and above clinical guidelines

Achieving effective guideline implementation reflects not only the people involved, but also their professional roles, their positions in the organisation and the epistemic communities to which they belong [10, 11]. Producers and users of evidence and guidelines often sit on different sides of the social, scientific and clinical boundaries. Therefore, they possess varying types of knowledge which may mean that they privilege evidence differently. This can make integration across these communities challenging [11]. Day-to-day decision-making by healthcare professionals requires the selection of many types of knowledge and evidence, situated within local, contextual and social circumstances [12]. For example, research knowledge coexists with the lived experience of patients and the macro healthcare initiatives and incentives from policymakers and regulatory bodies.

Preserving clinical autonomy and medical judgement by these professional groups is also recognised as important, particularly when guidelines challenge traditional practices [13, 14]. In this study, we examine guideline implementation in the context of real-life NHS practice. We investigate guideline implementation problems through the application of a range of qualitative methods to explore where, when and how evidence and knowledge were used in clinical decision-making in elective orthopaedic surgery.

### Guideline implementation in orthopaedic surgery

A mixed methods systematic review has identified various sources of evidence and knowledge which influence decision-making within orthopaedic surgery [15]. The findings revealed several factors which impact upon guideline implementation specific to the orthopaedic specialty. For example, compared to other clinical specialties, orthopaedic surgery represents a highly professionalised area of clinical work where elite communities of practice are strongly embedded [16]. There is a tendency towards decisive and authoritative patterns of decision-making, and surgeons are able to retain substantial autonomy over their work practices to resist external intervention [15].

Enhanced implementation of guidelines is an unexplored area of research in the highly professionalised, intensely networked group that is orthopaedic surgery. This study provides an in-depth understanding of decision-making using clinical guidance. It generates a unique perspective on the challenges of translating research into clinical practice and moves away from the approach of assessing compliance or auditing guideline uptake. A key part of understanding complex problems, such as where, when and how evidence and knowledge are used in practice, requires examining the values, beliefs and norms of individuals who are responsible for making decisions in context. This complements a broader investigation of an organisation’s capacity to support clinical guideline implementation and of other contextual factors within the healthcare sector which influence the use of knowledge and evidence in practice.

## Methods

We examined the implementation of an example of NICE guidance within orthopaedic surgery, to identify similarities and differences in the way this type of evidence is used in practice. In 2014, NICE released updated guidance on hip implants for total hip replacement for end-stage arthritis (see Additional file 1: Appendix SP1 for an example of the guideline recommendations) [17]. At the time of our study, it had been over a decade since the previous version of the guidance was disseminated to practitioners. Therefore, our study was ideally timed to investigate the implementation of this updated guidance in the NHS to facilitate more general exploration of guidelines.

Three comparative case studies were conducted, using qualitative methods with multiple levels of analysis [18]. The case studies were conducted in UK hospital Trusts in the midlands, north and south west of England (see Table 1). A hospital Trust is an organisation that provides secondary healthcare services to a locality within the English NHS system. The protocol for the entire study has been described in detail elsewhere [19]. Each case study traced the implementation of NICE guidance in practice to explore the understanding and use of evidence and knowledge in orthopaedic surgery. We selected cases to represent maximal variation in orthopaedic services in England [20]. For example, an orthopaedic department in a teaching hospital, one in a non-teaching hospital and a third in a designated academic orthopaedic department where staff members hold hybrid academic/clinical roles in both the hospital and affiliated University.

**Table 1:** Case study setting and participants

Descriptor	Case study A	Case study B	Case study C
Setting	Orthopaedic trauma centre	Small hospital Trust split between two geographical sites. Therefore the orthopaedic services were separated across two hospital buildings	Large orthopaedic department with specialist trauma centre which received national referrals for complex hip implant revision surgery
University link	Teaching hospital with a designated academic orthopaedic department located in a university owned building within the NHS hospital	None	Teaching hospital
Participants	A majority of surgeons held joint posts between the NHS and the same university department. The staff conducted clinical effectiveness and cost-effectiveness research, mainly national randomised controlled trials of various techniques and treatments within orthopaedic surgery	Surgeons provided general orthopaedic services to the local population supported by a designated group of allied health professionals	Surgeons in the teaching hospital held contracts with the NHS hospital. A minority of surgeons held honorary contracts with one of the four universities in the region, i.e. they were not from the same academic department

We followed the roadmap for a case study research developed by Eisenhardt [21] where each case study started as close as possible to the ideal of *no theory under consideration.* This method prevents any pre-selected theoretical perspectives limiting the data collection process [21]. The research was abductive in nature. We were informed by previous literature and theory but also by the data collected [22].

Data collection in the field allowed for concepts of interest to develop as the case studies progressed [23]. This flexible approach is a key feature of case study designs, which enabled us to adjust data collection processes to further investigate emergent themes and to take advantage of opportunities as they arose [24, 25]. For example, the importance of groups of surgical colleagues acting as communities of practice (as a potential theme) grew, as more data was collected and as case studies progressed in series. This enabled us to search for specific instances of surgeons in communities in the later cases and thus formed part of the data collection process.

Across the three cases, we sampled orthopaedic surgeons and Allied Health Professionals (AHPs) who conducted or facilitated joint replacement surgery, i.e., we sampled purposively aiming for heterogeneity of professional background, level of training and years in practice. Snowball sampling enabled us to follow direct recommendations from participants. We aimed to explore guideline implementation from all perspectives, so we also invited administrators and managers involved in guideline implementation and in the decisions made for patients undergoing hip replacement surgery.

### Data collection

We selected a combination of qualitative methods including document analysis, observation and interviews. One author (AG) was responsible for all data collection in each of the cases. Data collection remained systematic and transparent, and decisions were recorded in case summaries. We continued data collection until no new information was obtained and theoretical saturation within each case was reached [26]. Within each case, 3 months of observation took place between 1 December 2014 and 11 December 2015. Observations consisted of opportunistic shadowing involving, watching clinic and teaching sessions and attendance at planned operating sessions, particularly pre-theatre preparation time. Observations enabled informal discussions with surgeons and clinical staff and provided an opportunity to describe actions and decisions in real time. Each observation was recorded in a field journal using a predetermined template.

Document analysis involved collection of key organisational documents such as clinical pathways describing structured multidisciplinary plan of care and hospital protocols (see Table 2) [27]. The documents helped us to understand and frame intentions to change practice within the orthopaedic departments. Analysis of the documents enabled us to gain a wider understanding of the context within which decisions were made.

**Table 2:** Document type and quantity by case study site

Document type	Case A	Case B	Case C
Clinical pathways	5	3	6
Protocols	17	2	4
Meeting notes	7	5	11
Strategy documents	2	1	0
Quarterly and annual reports	14	18	17
Internal presentations	2	5	2
Sub-total	47	34	40
Total	121		

We interviewed 64 participants between December 2014 and December 2015. During the interviews, we sought to understand the approaches and beliefs of participants regarding knowledge and evidence, in order to reveal the strategies used by professionals when making decisions. Questions explored the extent of professionals’ beliefs regarding NICE and the involvement and impact of clinical guidance on surgical practice within their hospital. The open interview format enabled participants to expand on topics of interest freely. We set out to discover what professionals considered to be evidence and knowledge in practice, rather than focusing on any pre-existing definition which may have restricted the findings. Each interview was labelled with location and timing and an anonymised identification number.

A copy of the interview topic guide is presented in the Additional file 1: Appendix SP2. Table 3 displays the different professional groups interviewed (‘C’ clinical, ‘A’ allied health professionals and ‘M’ managers). To obtain a national perspective, we conducted eight key informant interviews with stakeholders from NICE, The Royal College of Surgeons, and Clinical Commissioning Groups.

**Table 3:** Participant numbers detailed by case study site and by professional group

Professional group	Case A	Case B	Case C	Key informant interviews
Clinical (C)	12	10	8	4
AHP (A)	4	5	6	2
Managers (M)	2	4	5	2
Sub-total	18	19	19	8
Total	64			

### Data analysis

As outlined in the roadmap method, all data were analysed, integrated and triangulated within case before comparative case analysis was undertaken across the cases. The three data sources were processed into text format to allow for thematic analysis through data familiarisation, coding and development of categories from codes [28]. The first stage of data analysis was conducted by one author (AG). Second round coding was performed jointly by all authors during two data analysis sessions. Coding differences were reconciled through discussion by all authors and refinement of first- and second-order codes was performed to generate categories and themes. The three types of qualitative data were integrated using the Pillar Integration Process [29]. This is a matrix integration technique for mixing data which has been collected using different methods.

We compared data across data collection methods. Triangulation between data collection methods facilitated the validation of cases as we searched for convergence among the multiple data sources. We triangulated data from the three data sources and interpreted them together to find common themes by eliminating overlapping areas and identifying areas of convergence [26]. We noted, for example, if a ‘guideline implementation process’ document in a hospital did not match with the data collected in the observations and interviews, this perhaps demonstrates that participants were not aware of this document or that it was not an important factor into decision-making. Each case was written up to provide narrative descriptions of the current situation at each hospital. This process was central to generating familiarity and insight [30]. It enabled us to see patterns in each case as they emerged and accelerated our cross case comparisons [21]. The goal of the cross case comparison was to search for further patterns in the data and to explore how these were represented or played out differently in the three cases.

## Results

**Fig. 1:**
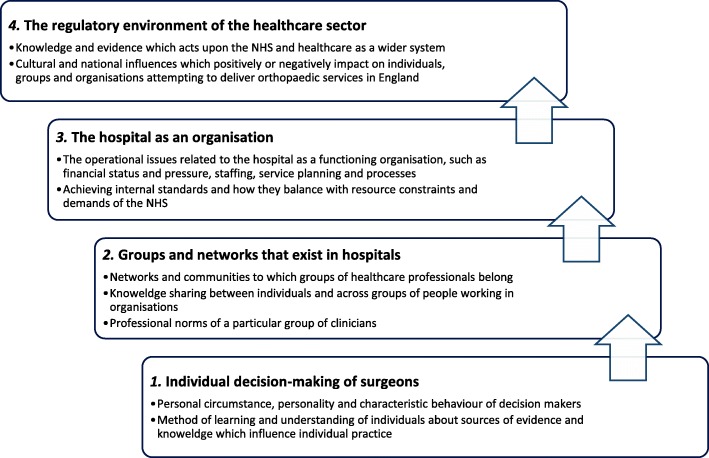
Visual representation of the four thematic findings which describe the influence of evidence and knowledge on decision-making

The overarching themes displayed in Fig. 1 represent broader narratives to describe the structural levels which influenced guideline implementation in elective orthopaedic practice. The evidence and knowledge of individual surgeons, groups of healthcare professionals, healthcare organisations and the regulatory environment interacted to produce the context for guideline implementation.

### Types of knowledge and evidence which influence the implementation of clinical guidance in elective orthopaedic surgery

**Fig. 2:**
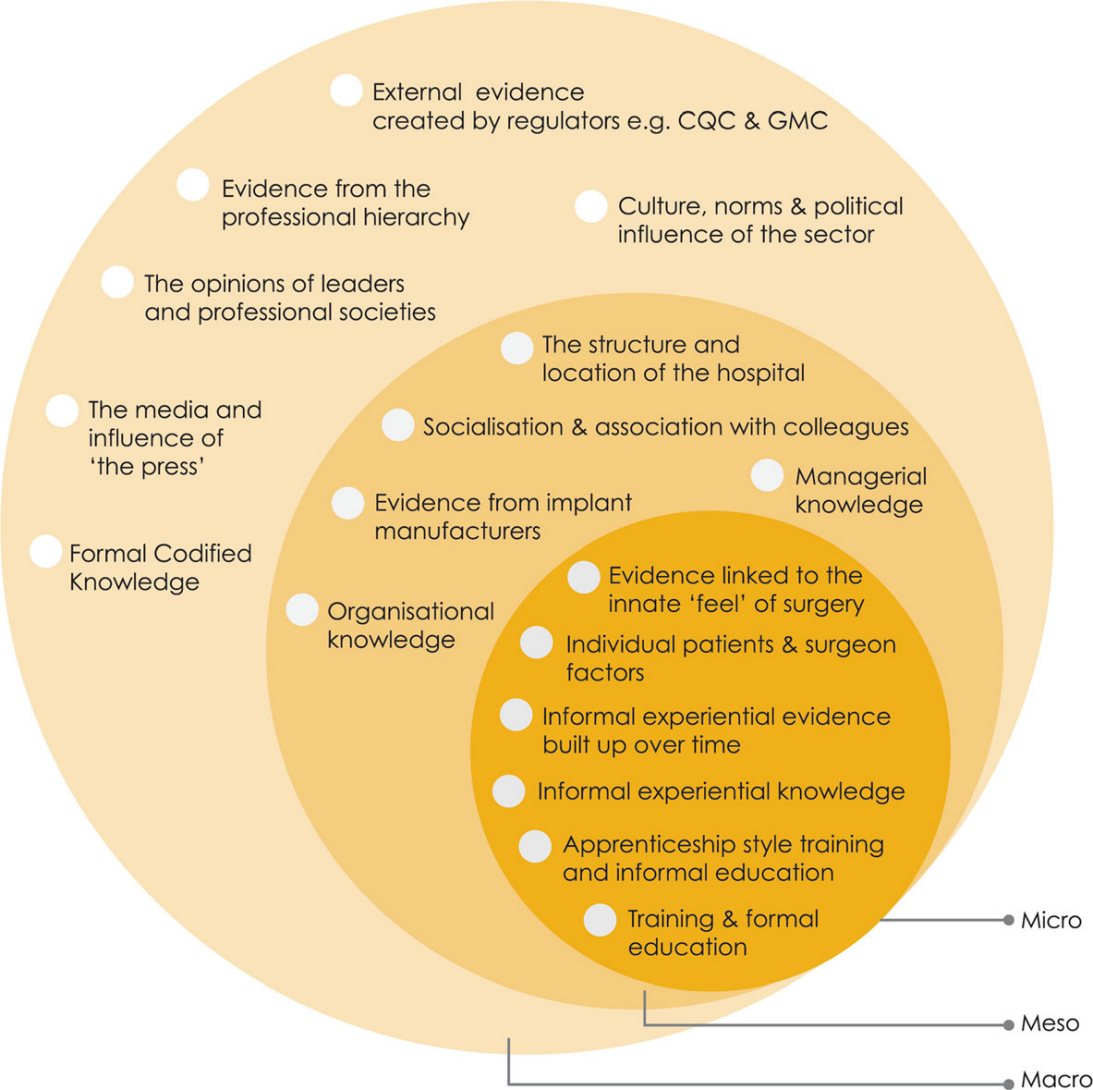
Summary of the of knowledge and evidence identified in case studies of guideline implementation in orthopaedic practice

Case studies revealed that a significant number and a diverse range of knowledge and evidence sources were used in decisions made by orthopaedic surgeons regarding hip replacement surgery. We characterised these sources into micro, meso or macro levels of influence, as displayed in Fig. 2. The sources of evidence and knowledge have been categorised this way to demonstrate the structural level at which they were enacted in practice [31]. The multi-level approach to synthesis helped to recognise the interdependence between the various levels. Micro knowledge and evidence that tended to influence individual decision makers, meso evidence and knowledge sources appeared to act at the level of the organisation, whereas macro knowledge and evidence existed in the higher domain of the wider healthcare environment. Narrative descriptions and an example of each source of evidence and knowledge are provided in Table 4.

**Table 4:** Narrative descriptions and an example of each source of evidence and knowledge depicted in Fig. 2

Evidence and knowledge types	Narrative description	Example from the data
Macro		
External evidence created by healthcare regulators, e.g. CQC and GMC	The wider delivery of healthcare in England is governed by the UK Health and Social Care Regulators such as the Care Quality Commission. In orthopaedics, surgeons have to be registered with the General Medical Council and with their Royal College. Regulators are responsible for ensuring that surgeons are included in an up-to-date registry of qualified doctors and practice according to established standards	An inspection report from the Care Quality Commission
The media and the influence of ‘the press’	The mass media (or press) is a diversified collection of resources who reach a large audience via mass communication	An article in a newspaper describing ‘good’ or ‘bad’ hip implants
The opinion of leaders and professional societies	An opinion leader was an eminent individual who had the ability to influence the opinion of the orthopaedic community on a subject matter for which they well known. The professional societies were larger organisations who represented the groups and sub-groups of surgeons	An opinion leader could be a principal investigator of a large clinical trial in orthopaedics. The professional societies were the British Orthopaedic Association and the Hip Society
Formal codified knowledge	Evidence or knowledge that is written down can be shared and is easy to access and available to the public	A NICE guideline or article published in a journal
Culture, norms and political influence of the sector	The standards and accepted way of practicing in the UK healthcare context. Including the public delivery of services and formal and informal methods in which healthcare is organised in the NHS	The hierarchical structure of the healthcare system. Political factors included strategies enforced by government and the medico-legal challenges to practice
Meso		
Managerial knowledge	Each hospitals’ business organisational processes which underpin day to day routines and capabilities of the Trust	NHS hospital resource issues such as time, cost and safety or quality of services
Organisational knowledge	An extension of managerial knowledge which has a wider structural emphasis. It is embedded in the processes of healthcare organisations and influences the behaviour of staff	A hospitals’ internal processes which are not written down. Anecdotally referred to as “the way we do things around here”
The structure and location of the hospital	The physical location of the hospital buildings and departments and the structure of the hospital wards	The number of elective orthopaedic theatres available to use
Evidence from implant manufacturing companies	Information that came directly from manufacturer’s representatives located in the hospital or indirectly through marketing	Leaflets about a hip implant from a manufacturer’s representative
Socialisation and association with colleagues	Knowledge that came from the inside and spread within the defined clinical group, in this case the orthopaedic community	Evidence of the outcome of a surgery from a colleague or knowledge that a mentor had passed on
Micro		
Informal experiential knowledge	Tacit knowledge that surgeons ‘know’ regarding how to behave and perform as an orthopaedic surgeon	Represents a surgeon’s lifetime’s work, and in turn their identity as a surgeon
Informal experiential knowledge built up over time	The tacit knowledge that surgeons ‘know’ which has built up over time working in the specific hospital but which can be difficult to describe	Knowing which colleague to refer a difficult case to when the surgeon does not have the specific expertise or experience
Evidence from the professional hierarchy	The layered social structure within the hospital which conceptualised the superior and inferior relationships between clinical staff	Described as the ‘clinical pecking order’ with the consultant surgeon at the top
Training and formal education	The training and formal education of healthcare professionals which are recognised through standard academic qualifications	A Master’s degree in Evidence-Based Medicine
Apprenticeship style training and informal education	Personal training which occurs during each working day with senior colleagues	Training gained through fellowship programmes and practice-based learning
Individual patient and surgeon factors	Characteristics of the patient or surgeon that influenced clinical practice decisions	Patients age or a surgeons years in practice
Evidence linked to the innate ‘feel’ of surgery	A description of the surgeon’s judgement, skill, craft and instinct	A surgeon not knowing exactly what will occur during an operation until they started the surgery and can see and feel the operation takes place

Evidence in the form of NICE guidance, i.e., *formal codified knowledge*, was just one of the 17 types of knowledge and evidence identified in our study. Therefore, the additional 16 sources of evidence, such as *the structure and location of the hospital* or *the opinions of leaders and professional societies*, influenced the uptake and use of clinical guidelines in orthopaedic surgery.

A determining factor of guideline implementation was how these knowledge and evidence types were amalgamated together in the different contexts of practice. The amalgamation process was flexible, adaptable and on-going, and therefore, the dominant source of evidence and knowledge in each case would often change. What was important to the decision-maker at one point in time was not always the presence or content of evidence-based guidelines (macro). It could equally be any other type of evidence or knowledge, interacting with, for example, a surgeon’s training and formal education (micro) or the contingencies of practice such as the structure of the hospital (meso).

### Distinct approaches to decision-making

Comparison across cases was essential to look beyond the initial impressions and see the findings through multiple lenses. We framed our cases according to their general approach to decision-making and how guidelines were implemented in each hospital. The differences in decision-making contributed to variation from the codified evidence contained in guidelines across all three cases. The analysis revealed that the individuals, groups and organisations in each case had a distinct approach to decision-making. The approach was dependent on how the 17 types of knowledge and evidence interacted, changed and were used in orthopaedic practice. Guideline implementation was contingent and mediated through the distinct contextual environments, which were subject to forces of the regulatory environment.

### Case A

Case A was an academic centre located within a trauma and orthopaedic department. More than the other two cases, case A appeared to have a positive view of the *formal codified knowledge* contained in clinical guidelines and what guidelines set out to achieve in the healthcare sector. The surgeons working in case A took a population perspective on clinical decisions. The *culture, norms and political influence of the sector* acted on case A in a positive fashion, as surgeons working here valued the processes and aims of generating clinical guidelines and the goals of NICE as an organisation. Surgeons were accustomed to answering questions using a larger population frame of reference and suggested that they “may subconsciously be following NICE guidance” (Junior surgeon) as it was indoctrinated in the *organisational knowledge* and processes. One surgeon states that “NICE guidelines help you look at cost effectiveness and evidence a lot more than you would think about in normal daily practice as an individual orthopaedic surgeon” (Consultant surgeon).

Case A had the most advanced and formalised guideline implementation processes i.e., their *managerial knowledge*. This was reflected in case A’s extensive protocol documentation compared to the other two cases (17 versus 4 and 2). Each protocol was linked to a piece of clinical guidance or an internal evidence summary which had been produced by medical librarians. The implementation process was described by the Chair of the Guideline Committee:

We started monitoring NICE implementation in the trust in 2011/12 when we set up the NICE implementation group…when NICE issues their guidance, I forward a list to the NICE administrator who would send it out to lead clinician in orthopaedics…If it’s a clinical guideline, or quality standard…there’s recommendations in there, and a form of a baseline assessment with the recommendations in…they (the clinician) have to indicate if we’re compliant or not. (Hybrid surgeon)

The surgeons in case A demonstrated a distinctly different trend in their confidence and appreciation of clinical guidance (*individual surgeon factors*). We consider that this was due to their departmental focus on research (*apprenticeship style training*) and academic output developed through *training and formal education* in Evidence Based Medicine (EBM), and beliefs regarding the importance of NICE and the EBM approach. A quote from a surgeon illustrates this:

*“We do try as much as possible to follow the basis of evidence...So the typical patients would be where we would need to use evidence. There is not much of a dilemma about someone with end stage osteoarthritis as the guidance shows the pathway that they should take.” (Consultant surgeon)*

The distinct approach to decision-making in case A was ‘pragmatic EBM decision-making’, where the traditional approach to evidence-based decision-making recommended in clinical guidelines was suitable, but not sufficient for their practice. Surgeons in case A acknowledge traditional EBM and focused on pragmatic EBM. This practice-based approach to EBM considered the important point that knowing what to do, how to do it, and the likely outcome of an orthopaedic intervention described in NICE guidelines were only part of the knowledge picture which had to fit into the ever changing context of practice. However, *external evidence created by regulators* and *managerial* and *organisational knowledge* could limit the behaviour and decisions of surgeons. For example, surgeons working here were restricted in the hip replacement implants, they could select based on cost and procurement contracts (meso) and thresholds established by the Orthopaedic Device Evaluation Panel in the UK (macro).

### Case B

Case B was a small hospital Trust split between two geographical sites. Case B demonstrated an ‘“it depends” approach to decision-making produced by the binary characteristics of the Trust’; this was bought about by *the structure and location of the hospital.* The binary characteristics reflect the two distinct hospital locations in case B. By name, the hospitals were one Trust; however, the day-to-day operations and decision-making practices were separated and distinct. Each hospital location in case B had their own way of doing things *(organisational knowledge)* and staff acted protectively to maintain them. The ‘it depends approach’ signifies the participants views that what happened in practice depended on which of the two case B hospital sites they were located at when the decision was made.

In case B, *external evidence created by regulators* was not regarded as important. *Formal codified evidence* in NICE guidelines was not valued by the clinical staff. Guidelines were often considered in a negative light and considered to be the responsibility of hospital administrators and managers (i.e., *managerial knowledge*). Described by a hospital board member below:

*“I think NICE guidance is very much just seen as another layer of administration for clinicians. If no one’s looking at whether you’re following NICE guidance or not they just sit on a shelf, unless you have a very active team of clinicians who take this on board. But that’s not a consistent.” (Hospital board member)*

The negative value attached to NICE and guidelines was echoed by the surgeon in the quote below, in which they demonstrate the importance of *socialisation and association with colleagues* when making clinical decisions:

*“NICE are not being proactive enough, I would say, in terms of making recommendations on prostheses and they could do a lot more. There is very little sort of robust evidence to guide practice so you rely on other peoples’ anecdotal experience and normal practice to help guide what, what works and what doesn’t” (Consultant surgeon)*

Within the organisational processes, guidelines were not considered an important part of practice in case B. Compliance to NICE guidelines as *evidence of external regulation* did not appear to be valued and hence implementation was haphazard, as described by a hospital administrator:

I send (guidance) out to the General Manager and then they will send it to the most appropriate person. We used to meet to discuss if we were compliant…but now we send a questionnaire out…so they have to do is tick ‘Yes’ and ‘No’ but they do not always respond. (Administrator)

What mattered for implementation in case B was the dynamics of organisational change and leadership enacted through evidence from the *professional hierarchy* in the two orthopaedic departments within the Trust. The *individual surgeon factors* and the differing characteristics and processes of the two groups of surgeons headed up by *opinion leaders* meant that guideline implementation across the organisation was difficult. The distinct groups struggled to work together and share *organisational knowledge* because the specific contexts that clinicians were socialised into differed. For example, *evidence from implant manufactures* carried greater weight in one location compared to the other. The *socialisation and association with colleagues* reflected the behaviour and norms that guided or regulated the action and decision-making of individuals at case B.

### Case C

Finally, case C was an orthopaedic department located in a teaching hospital. In case C, ‘socialised decision-making was prominent and evidence was discretionary’. The orthopaedic department in case C was closed to the influence of administrators and *managerial knowledge* emerging from elsewhere in the Trust. They were also resistant to pressure from external policymakers and *evidence from regulators*. This divide across professional boundaries compounded by the *professional hierarchy* and *cultural norms of the sector* made guideline implementation challenging. Nevertheless, knowledge and evidence generated through *managerial and organisational knowledge* was present in the wider hospital organisation:

We have standard operating procedures for NICE, which encourage [surgeons] to write their own action plan (Trauma & Orthopaedic Manager)

However, observations of practice revealed that *managerial and organisational knowledge* enacted through processes attached to NICE guidelines and governance belonged in the managerial and administrative domain, not within surgical staff. Surgeons reported having “never seen” the organisation’s NICE process (Consultant surgeon). This is reflected in the observation note below:

NICE was rarely noted as an influential factor in the day-to-day activities of surgeons. Surgeons I spoke to were unaware their hospital had a NICE process, they would respond “do we have one?” “I’ve never seen it”. (Observation note)

Clinical practice decisions were made using knowledge and evidence gained through *socialisation and association with colleagues.* Surgeons possessed resilient *experiential knowledge built up over time* as the majority had been working in this hospital for their entire careers and were relatively separate from ‘outside’ knowledge. *Formal codified evidence* in clinical guidelines had to compete with the complex social systems that existed in the hospital. For example, case C was a referral centre which specialised in performing complex hip replacement revision surgery. In this context, clinical guidelines appeared to be less important because the approaches, techniques and implants needed to perform hip revisions were specialist (*the innate feel of surgery*) and therefore not included in guideline recommendations. One surgeon noted “NICE, is irrelevant. They don’t tell me anything I NEED to know” (Consultant surgeon). The specialist surgeons working in case C referred to a standard hip replacement operation as “boring” and, hence the work of other surgeons “on the treadmill” who are outside of their community or social system (Consultant surgeon). In this sense, guidelines were not valued or applicable to their specialist work. The *informal experimental knowledge* and *informal experiential evidence built up over time* appeared to take precedence in clinical decision-making processes and therefore restricted guideline implementation of guidelines for this group of surgeons. These dominate types of knowledge and evidence were grounded in the experience of surgical work in practice and legacy knowledge about the organisational functions which surgeons developed as a consequence of working there for a long period of time.

## Discussion

In orthopaedic surgery, clinical guidelines are an important part of practice as they not only help guarantee safety and encourage quality improvement but also ensure that NHS resources are used appropriately [32]. The aim of this study was to identify where, when and how evidence and knowledge are used in healthcare decision-making in orthopaedic surgery and how variation in evidence and knowledge contributes to differences in the implementation of clinical guidance in practice. Previous scholars have counted and categorised evidence using broader taxonomies, which describe knowledge as individual, group, tacit or explicit [33–35]. Others emphasise the role of the person in the activity to distinguish between action, doing and practice, and knowledge facts and processes [36]. It is important to highlight that what is considered evidence and knowledge is highly contested and influenced by the environment in which it is used [10].

In fulfilling our aim, we discovered 17 different types of knowledge and evidence which were used in orthopaedic surgery. During our case comparison, it became clear that the dynamic selection, combination and use of each type of knowledge and evidence influenced the implementation and use of clinical guidance in practice. At the time of study, none of the cases could definitively provide evidence to demonstrate that they were accessing, using and monitoring guideline recommendations. We examined implementation of guidelines by individual surgeons, groups of surgeons and the Trusts as a whole. Interestingly, implementation could differ between these levels.

In the context of orthopaedic surgery, the process of privileging different types of knowledge and evidence in the context of surgery resulted in three distinct approaches to decision-making in orthopaedic surgery. These include ‘pragmatic EBM decision-making’ (case A), where NICE guidelines failed to deliver all the knowledge and evidence needed to make clinical decisions when organisational context restricted surgeon choice. An ‘“it depends” approach produced by the binary characteristics’ of case B linked to the professional hierarchy of surgeons working in separate geographical locations and ‘socialised decision-making’ where evidence was discretionary due to the strong influence of socialisation and informal knowledge sharing between surgeons in case C.

The similarities and differences between the three approaches generate key contextual dimensions specific to guideline implementation in orthopaedic surgery. These reflect the relationship between guideline implementation and knowledge and evidence that is actually used in orthopaedic practice. Orthopaedic surgeons in our study held ambivalent or negative attitudes towards clinical guidelines. They did not privilege this *formal codified evidence* because it originated outside of orthopaedics and did not contain the micro sources of knowledge and evidence (e.g., experience, training, individual characteristics, and the innate feel of surgery) that were considered more important for surgeons’ decision-making. However, the *culture and norms* of EBM, identified in case A, demonstrate that it was possible to positively influence guideline implementation. Consideration of the power of professional hierarchies is vital, as surgeons working at the top of the hierarchy, such as clinical leaders can restrict or diminish the influence of mangers and policymakers. Organisational constraints linked to financial restrictions, regulation and procurement-influenced implementation and a lack of focus on clinical guidelines in orthopaedic practice. The presence of organisational processes and protocols could not ensure what guidelines were valued and used. What was more important is what evidence and knowledge transferred from surgical colleagues and professional societies.

It is likely that many of the 17 types of knowledge and evidence identified in this research would not have been discovered without the structured, comparative case study approach used in our study. One of the aims of our research was to go beyond reports in the previous guideline implementation literature [3–9, 37]. It is significant that a large number and diverse range of knowledge and evidence types acting across the entire domain of healthcare emerged from the three case studies of NHS practice. Previous research has identified a considerable number of barriers and facilitators to the implementation of clinical guidelines but often they are too generic or limited in scope, for example, the difficultly of engaging individual clinicians or problems with process and resource issues within hospitals [7, 13, 14].

We have demonstrated that guideline implementation and subsequent evidence-based practice were not always possible or preferable in the three cases. Comparative case study analysis revealed dynamic contextual differences and variation in practices and processes between the three hospitals. For example, some surgeons had strict limits placed on the orthopaedic implants they could order within their hospital. This restricted their implant decisions. However, this varied across the cases and differences were found in implant selection practices and processes. This variation had a direct impact on the uptake and use of evidence in practice and demonstrates the problems of effectively implementing standardised guidance in surgery. One of the aims of clinical guidance is to reduce unjustified variation in practice, but we found that this was not achievable or appropriate in all contexts.

Our findings encourage more research into how to engage individuals and groups of healthcare professionals to consider the content of clinical guidelines and how it might add to their decision-making processes. The overarching view that guidelines are the responsibility of managers and administrators demonstrates a lack of ownership of the guideline in general in orthopaedics. Also, in the context of orthopaedics, our findings provide insight into approaches to knowledge mobilisation targeted at communities of practice as an area for investigation and improvement. Improvements in the uptake and use of NICE guidance in orthopaedic practice will require the development, presentation and dissemination of evidence-based guidelines in surgery to be better tailored to the orthopaedic community.

### Strengths and limitations

The key strength of our research is the use of case studies to examine the context of guideline implementation. In achieving our aim, we were able to discover and explain the gap in implementation of clinical guidelines in orthopaedics. Uptake and use of guidelines, even when grounded on the findings of empirical research including gold standard randomised controlled trials, were not guaranteed in practice. This suggests that well-developed guidelines are necessary but not sufficient to achieve the goals of policymaking organisations such as NICE and Scottish Intercollegiate Guidelines Network (SIGN). Case study methods allowed us to describe the decision-making context in detail. This method has provided a greater depth of description of the evidence and knowledge sources than have been outlined in previous literature, for example, expanding the tacit-explicit-group-individual categorisations*.* The way in which evidence and knowledge interacted with context produced variation in the extent to which guidelines are implemented and used in orthopaedics.

A second strength of our research is the use of multiple data sources (interviews, observations and document analysis) to study the same phenomena. The combination of methods facilitated us to overcome the weaknesses that emanate from selecting a single method to study the complex process and practice of guideline implementation [38]. We triangulated data to enhance the credibility of our analysis and findings. When data from one source substantiates a pattern from another, the findings are stronger and better substantiated [26]. Comparison across our three comparative cases enabled us to generate a more sophisticated understanding of the data we collected [22, 23].

Our study has limitations. The direct observation of healthcare professionals in their practice may have driven a change to ‘good’ or ‘better’ behaviour by participants; a phenomenon known as observer bias [39]. To ensure the quality and rigour of our data, we extended our access and observation as much as possible, whilst also conducting crosschecks and validation during interviews and between different individuals and professional groups. We sampled three hospitals from the population of 135 hospital Trusts in England which deliver hip replacement services [40]. Although the sample was small, we aimed to achieve a broad representation of the types of elective orthopaedic services available in England and aimed to produce in-depth rather than a breadth in our case study design and data.

### Policy and practice implications

Over the last 20 years, there have been significant changes in the way policy-making organisations such as NICE create and disseminate guidance to improve health and social care. What appears to have remained constant is the way in which codified knowledge in guidance is produced with an assumption that a linear ‘push’ fashion will ensure that it is received and acted on by clinicians working in healthcare organisations. The findings of our study reconfirm and extend our knowledge of the limits of this approach for improving the use of guidance and for reducing variation in practice for orthopaedics.

In this study, we have raised the issue of whether NICE guidelines are ever likely to be appropriate for the field of orthopaedics. This is due to the wide range of knowledge and evidence identified as influential to decision-making, coupled with the differences in guideline implementation across the structural levels. However, we do not consider that the evidence contained in guidelines is inappropriate. Instead, the ways in which knowledge and evidence were privileged differently by individual practitioners, groups of surgeons and organisations meant that guidelines were rarely accessed as a beneficial evidence source. Surgeons were not concerned about what guidelines recommended. What was important was their definition of knowledge and evidence and how this interacted with understandings of knowledge and evidence in their group and wider organisation. In this study, ‘one size’ guidance could never ‘fit all’ the surgeons’ requirements and therefore, the guidance had limited value in their specific circumstances.

Nevertheless, evidence in guidelines represents best practice, and NICE and SIGN must produce recommendations for healthcare. We have provided evidence to suggest that the current modes of transfer and implementation are ineffective. Changes could be made to the process of guideline creation, dissemination or even regulation to move towards effective knowledge mobilisation. For example, a more inclusive process of involving clinicians in guideline development would be welcomed. The current system relies on clinicians being aware of guideline updates, rather than being enabled to actively volunteer their contributions. Targeting the orthopaedic community through professional meetings, networks and clinical leaders would communicate the need for involvement and increase discussion about new guidelines in contrast to the current process of one-way dissemination by policymakers.

Regulation was a valuable mechanism as it achieved a desired outcome for achieving targets and controlling the behaviour of the surgeons. However, moving towards regulation as the norm did not appear to be a desirable option for most of the professional groups in this study. Knowledge and evidence from external regulators did not hold the same positive status achieved by the knowledge and evidence emanating from colleague and professional networks. Restricting the discretion and authority of clinical professionals by increasing regulatory power would not be recommended. Instead, interventions which take advantage of the positive knowledge mobilisation between orthopaedic colleagues are encouraged, as they may improve the sharing of evidence-based practice.

Improvements need to be made to how healthcare professionals working in hospitals see and think about evidence from guidelines in combination with other knowledge and evidence sources. Improvement interventions are required to help users of guidance identify ‘where they are at’ in their decision-making processes. Clinical guidelines were often unable to provide a solution to a decision problem; therefore, it is important to understand what other types of evidence and knowledge are available or used by others. Practitioners need to acknowledge the difference between certain types of knowledge as positive or negative to patient care. Where possible, those working in healthcare should focus on reducing undesirable types of evidence and knowledge present in their organisation. This could be an area for improvement work. However, surgeons in this study were often unaware of or ambivalent about the consequence of their decisions because processes were not open, transparent or subject to feedback loops.

Healthcare practitioners could take a more transparent approach in understanding the evidence that is driving their decisions and how guidelines may fit into the picture. This will facilitate practitioners in deciding whether guideline recommendations are appropriate in their context. If not, other knowledge sources such as clinical experience could take precedence and be shared, explained and understood, rather than frowned upon by managers and administrators and recorded as an organisational risk. Practice-based knowledge was rarely shared between the professional groups in this study. Encouraging open decision-making processes might enable those on all sides of knowledge boundaries to understand and accept why certain options are chosen and actions are taken, especially if they vary from guideline recommendations.

## Conclusion

The research aimed to explore guideline implementation through the application of comparative case studies which investigate the use of NICE clinical guidelines in decisions made in elective orthopaedic surgery in the NHS. The results of our study highlight the range of complex and competing sources of evidence and knowledge which influence the work practices of healthcare professionals. Case study analysis revealed three distinct styles of orthopaedic practice which represent the ways in which 17 types of knowledge and evidence were used during decision-making. The way in which evidence and knowledge were selected and used impacted on how guidelines were implemented in the orthopaedic speciality. Findings from the case comparison reflect the complexity of evidence-based decision-making in the highly professionalised organisationally regulated context of surgery. Our results could be used to guide the development of implementation interventions that are grounded in the findings of this study. New approaches to implementation need to appreciate and incorporate the diverse range of knowledge and evidence which influences clinical decisions in orthopaedics and to take account of the changing contextual situations in which decisions need to be made.

## Additional file

Additional file 1: An example of a NICE guidance recommendation. Technology appraisal guidance [TA304] [17]. Total hip replacement and resurfacing arthroplasty for end-stage arthritis of the hip. Interview topic guide. (DOCX 15 kb)

## Abbreviations

AHPs: Allied Health Professionals (including Occupational Therapists, Operating Department Practitioners, Physiotherapists, Nurses and Healthcare Assistants)
EBM: Evidence-based medicine
NHS: National Health Service
SIGN: Scottish Intercollegiate Guidelines Network

## References

[1] NICE. About. 2017. http://www.nice.org.uk/about. Accessed on 6 Dec 2017.

[2] SIGN. Who we are. 2017. http://www.sign.ac.uk/who-we-are.html. Accessed on 6 Dec 2017.

[3] Lowson K, Jenks M, Filby A, Carr L, Campbell B, Powell J (2015). Examining the implementation of NICE guidance: cross-sectional survey of the use of NICE interventional procedures guidance by NHS Trusts. Implement Sci.

[4] Sheldon T, Cullum N, Dawson D, Lankshear L, Lowson K, Watt I, West P, Wright D, Wright J (2004). What’s the evidence that NICE guidance has been implemented? Results from a national evaluation using time series analysis, audit of patients’ notes, and interviews. BMJ.

[5] Weng Y-H, Kuo K, Yang C-Y, Lo H-L, Chen C, Chiu Y-W (2013). Implementation of evidence-based practice across medical, nursing, pharmacological and allied healthcare professionals: a questionnaire survey in nationwide hospital settings. Impact Sci Soc.

[6] Francke A, Smit M, de Veer A, Mistiaen P (2008). Factors influencing the implementation of clinical guidelines for health care professionals: a systematic meta-review. BMC Med Inform Decis Mak.

[7] Fitzgerald A, Lethaby A, Cikalo M, Glanville J, Wood H. Review of systematic reviews exploring the implementation/uptake od guidelines. York Health Economics Consortium. 2014. https://www.nice.org.uk/guidance/ph56/evidence/evidence-review-2-431762366. Accessed 8 Jan 2018.

[8] Royal College of Physicians. Implementing NICE public health guidance for the workplace: a National Organisational Audit of NHS Trusts in England. 2015. https://www.rcplondon.ac.uk/projects/outputs/implementing-nice-public-health-guidance-workplace-2013-round-2. Accessed 8 Jan 2018.

[9] Platt C, Larcombe J, Dudley J, McNulty C, Banerjee J, Gyoffry G, Pike K, Jadresic L (2015). Implementation of NICE guidance on urinary tract infections in children in primary and secondary care. Acta Paediatr.

[10] Gkeredakis E, Swan J, Powell J, Nicolini D, Scarbrough H, Roginski C, Taylor-Phillips S, Clarke A (2011). Mind the gap: understanding utilisation of evidence and policy in health care management practice. J Health Organ Manag.

[11] Currie G, El Enany N, Lockett A (2014). Intra-professional dynamics in translational health research. The perspective of social scientists. Soc Sci Med.

[12] Davies HTO, Powell AE, Nutley SM (2015). Mobilising knowledge to improve UK health care: learning from other countries and other sectors—a multimethod mapping study. Health Serv Deliv Res.

[13] Denny K (1999). Evidence-based medicine and medical authority. J Med Humanit.

[14] Timmermans S (2005). From autonomy to accountability: the role of clinical practice guidelines in professional power. Perspect Biol Med.

[15] Grove A, Johnson R, Clarke A, Currie G (2016). Evidence and the drivers of variation in orthopaedic surgical work: a mixed method systematic review. Health Syst Policy Res.

[16] Ferlie W, Wood M, Fitzgerald L (1999). Some limits to evidence-based medicine: a case study from elective orthopaedics. Qual Health Care.

[17] NICE Technology Appraisal (TA304). Total hip replacement and resurfacing arthroplasty for end-stage arthritis of the hip. NICE. 2014. https://www.nice.org.uk/guidance/ta304. Accessed 30 Nov 2017.

[18] Stake R (1995). The art of case study research.

[19] Grove A, Clarke A, Currie G (2015). The barriers and facilitators to the implementation of clinical guidance in elective orthopaedic surgery: a qualitative study protocol. Implement Sci.

[20] Sandelowski M (1986). The problem of rigor in qualitative research. Adv Nurs Sci.

[21] Eisenhardt KM (1989). Building theories from case study research. Acad Manag Rev.

[22] Mantere S, Ketokivi M (2013). Reasoning in organization science. Acad Manag Rev.

[23] Parlett M, Hamilton D, Glass G (1976). Evaluation as illumination: a new approach to the study of innovatory programs. Evaluation studies review annual.

[24] George AL, Bennett A (2005). Case studies and theory development in the social sciences.

[25] Yin R (1984). Case study research.

[26] Creswell J, Clark V (2010). Designing and conducting mixed methods research.

[27] Kinsman L, Rotter T, James E, Snow P, Willis J (2010). What is a clinical pathway? Development of a definition to inform the debate. BMC Med.

[28] Braun V, Clarke V (2006). Using thematic analysis in psychology. Qual Res Psychol.

[29] Johnson R, Grove A, Clarke A. Pillar integration process: a technique to integrate data in mixed methods research. J Mixed Methods Res. 2017:1–20. 10.1177/1558689817743108.

[30] Pettigrew AM (1990). Longitudinal field research on change: theory and practice. Organ Sci.

[31] Pope C, Robert G, Bate P, Le May A, Gabbay J (2006). Lost in translation: a multi-level case study of the metamorphosis of meanings and action in public sector organizational innovation. Public Adm.

[32] NICE. The NICE way. Lessons learned from the National Institute for Health and Care Excellence. 2014. https://www.nice.org.uk/news/article/using-nice-s-approach-to-base-policy-decisions-on-evidence-could-help-save-billions. Accessed 30 Nov 2017.

[33] Cook S, Brown J (1999). Bridging epistemologies: the generative dance between organizational knowledge and organizational knowing. Organ Sci.

[34] Nonaka I (1994). A dynamic theory of organizational knowledge creation. Organ Sci.

[35] Lam A (2000). Tacit knowledge, organizational learning and societal institutions: an integrated framework. Organ Stud.

[36] Orlikowski W (2002). Knowing in practice: enacting a collective capability in distributed organizing. Organ Sci.

[37] Berry K, Haddock G (2008). The implementation of the NICE guidelines for schizophrenia: barriers to the implementation of psychological interventions and recommendations for the future. Psychol Psychother.

[38] Dixon-Woods M, Agarwal S, Young B, Jones D, Sutton S (2004). Integrative approaches to qualitative and quantitative evidence.

[39] Snyder ML, Frankel A (1976). Observer bias: a stringent test of behavior engulfing the field. J Pers Soc Psychol.

[40] NHS Confederation. NHS statistics, facts and figures. 2017. http://www.nhsconfed.org/resources/key-statistics-on-the-nhs. Accessed on 6 Dec 2017.

