# Supplementary material for: How are evidence and knowledge used in orthopaedic decision-making? Three comparative case studies of different approaches to implementation of clinical guidance in practice
Source: Implement Sci. 2018 May 31;13:75. doi: 10.1186/s13012-018-0771-4 (PMC5984395; doi:10.1186/s13012-018-0771-4)
Supplement: Supplementary file 1 — An example of a NICE guidance recommendation. Technology appraisal guidance [TA304] [17]. Total hip replacement and resurfacing arthroplasty for end-stage arthritis of the hip. Interview topic guide. (DOCX 15 kb) [file 13012_2018_771_MOESM1_ESM.docx]

**Additional file 1**

**Appendix**

**SP 1.** An example of a NICE guidance recommendation. Technology appraisal guidance [TA304] [18]. Total hip replacement and resurfacing arthroplasty for end-stage arthritis of the hip

The NICE guidance recommendations in TA304 read:

*“Prostheses for total hip replacement and resurfacing arthroplasty are recommended as treatment options for people with end-stage arthritis of the hip only if the prostheses have rates (or projected rates) of revision of 5% or less at 10 years.”*

**SP 2.** Interview topic guide

1. Descriptions of the general approach to practice and how clinicians approach treatment decisions
   1. What strategies are used by professionals when making clinical decisions?
   2. Questions to understand surgeons and staff approach to clinical evidence
2. Discussion about the sources of evidence and knowledge that influence practice in general
   1. Questions to gather professional narratives to understand the influence of pre-existing regulatory practice
3. Participants’ beliefs and experiences of using or having contact with clinical guidance (NICE in particular)
   1. Questions to explore the implementation of clinical guidance from the individual’s perspective
   2. What they consider the extent of their involvement and impact upon surgical practice
4. Participants’ views regarding how EBM and clinical guidelines could be better mobilised into practice
   1. Exploration of the importance, implementation and integration of clinical guidance in practice
